# Supplementary material for: Comparative Genomics and Physiological Characterization of Two Aerobic Spore Formers Isolated from Human Ileal Samples
Source: Int J Mol Sci. 2022 Nov 29;23(23):14946. doi: 10.3390/ijms232314946 (PMC9739757; doi:10.3390/ijms232314946)
Supplement: Supplementary file 1 [file ijms-23-14946-s001.zip › Supplementary_Material.pdf]

Supplementary Material

Table S1. Genes coding for vitamins found in SF106 and percentage of identity with proteins encoded by the reference strain *B. subtilis* 168.

**Vitamin**	**Gene name**	**Protein ID of *B. subtilis* 168**	**protein identity (%)**	**Target protein**
**B1**	*tenA*	CAB09525.1	99.6	SF106_1808
	tenI	QJF45101.1	99.5	SF106_1807
	thiO	QJF45100.1	100	SF106_1806
	thiS	QJR48708.1	100	SF106_1805
	thiG	NP_389051.1	100	SF106_1804
	thiF	NP_389052.1	100	SF106_1803
	thiD	QJF45097.1	100	SF106_1802
	ykoF	AQR85506.1	100	SF106_1635
	ykoE	AQR85505.1	100	SF106_1636
	ykoD	AQR85504.1	100	SF106_1637
	ykoC	AQR85503.1	100	SF106_1638
	ywbI	NP_391710.1	100	SF106_3767
	thiM	NP_391709.1	100	SF106_3768
	thiE	NP_391708.1	100	SF106_3769
	thiC	QJF41345.1	100	SF106_2092
**B2**	*ribD*	QJF43944.1	100	SF106_0787
	ribE	NP_390208.1	100	SF106_0788
	ribBA	AQR86551.1	100	SF106_0789
	ribH	AAA67484.1	100	SF106_0790
	ribT	QJF43948.1	100	SF106_0791
	nupP	QJF43123.1	100	SF106_2704
	nupQ	QJF43122.1	99.7	SF106_2703
	ribF	AQR85861.1	99.7	SF106_1280
**B5**	*ilvB*	NP_390709.1	100	SF106_0268
	ilvH	NP_390708.2	99.4	SF106_0269
	ilvC	NP_390707.1	100	SF106_0270
	leuA	NP_390706.1	99.81	SF106_0271
	panB	ABN11531.1	100	SF106_0873
	panC	NP_390123.1	100	SF106_0874
	panD	NP_390122.1	100	SF106_0875
	ilvD	NP_390070.2	100	SF106_0929
	coaA	NP_390257.2	99.7	SF106_0737
	coaX	NP_387951.2	99.6	SF106_3930
	panE	NP_389394.1	100	SF106_1436
	panE	NP_390070.2	100	SF106_0929
	*panS**	CAF1791342.1	99.4	SF106_1002
**B6**	*pdxT*	QJF42193.1	100	SF106_3980
	pdxS	NP_387893.1	100	SF106_3981
	gabR	QJF41824.1	100	SF106_2998
**B8**	*bioW*	NP_390902.3	100	SF106_0075
	bioA	QJF43255.1	100	SF106_0076
	bioF	QJF43256.1	99.7	SF106_0077
	bioD	QJF43257.1	99.6	SF106_0078
	bioB	QJF43258.1	100	SF106_0079
	bioI	NP_390897.1	99.8	SF106_0080

**B9**	*folB*	NP_387959.1	100	SF106 3922
	folP	NP_387958.1	100	SF106 3923
	pabC	NP_387957.1	100	SF106 3924
	pabA	NP_387956.1	100	SF106 3925
	pabB	NP_387955.1	100	SF106 3926
	folK	NP_387960.1	100	SF106_3921
	folA	NP_391956.1	100	SF106_3512
	dfrA	NP_390064.1	100	SF106_0935
	mtrA	ABN13184.1	100	SF106_0838
	mtrB	QJF43994.1	98.7	SF106_0839
**K2**	*menF*	NP_390961.1	100	SF106_0016
	mend	NP_390960.1	99.8	SF106_0017
	menH	NP_390959.1	100	SF106_0018
	menB	NP_390958.1	100	SF106_0019
	menE	NP_390957.1	100	SF106_0020
	menC	NP_390956.1	100	SF106_0021
	hepS	ABN13182.1	97.6	SF106_0840
	menG	NP_390156.1	97.9	SF106_0841
	hepT	ABN13180.1	99.1	SF106_0842
	*menI**	AOR99459.1	96.8	SF106_2693
	menA	NP_391728.1	100	SF106_3749
**Lipoic Acid**	*lipM*	QJF43820.1	100	SF106_0658
	lplJ	AQR85188.1	100	SF106_1953
	lipA	QJF41942.1	100	SF106_2875
	lipL	NP_391644.1	100	SF106_3835

"*" the strain of *B. subtilis* is not indicated

Table S2. Genes coding for vitamins found in SF174 and percentage of identity with proteins encoded by the reference strain *A. clausii* ENTPro.

	**gene name**	**protein ID of *A. clausii* ENTPro**	**protein identity (%)**	**Target protein**
**B1**	*thiD*	WP_035204020.1	100	SF174_1998
	thiE	WP_011246576.1	100	SF174_2002
	thiD	WP_035203906.1	99.6	SF174_2111
	thiM	WP_011247754.1	99.6	SF174_4166
**B2**	*ycsE*	WP_011247403.1	100	SF174_0272
	ycsE	WP_011248796.1	100	SF174_2249
	ycsE	WP_011246931.1	100	SF174_3980
	ribF	WP_081427623.1	100	SF174_0617
	ribH	WP_011246656.1	100	SF174_1917
	ribBA	WP_011246655.1	100	SF174_1918
	ribE	WP_011246654.1	100	SF174_1919
	ribD	WP_035205795.1	100	SF174_2223
	coaE	WP_011247549.1	100	SF174_0124

**B5**	*ilvD*	WP_0112475489.1	100	SF174_0186
	ilvG	WP_011247488.1	100	SF174_0187
	ilvH	WP_035201833.1	100	SF174_0188
	ilvC	WP_011247486.1	100	SF174_0189
	coaA	WP_035201761.1	100	SF174_0293
	coaD	WP_011247220.1	100	SF174_0462
	panE	WP_011247211.1	99.7	SF174_0471
	coaBC	WP_035201526.1	100	SF174_0520
	coaBC	WP_035203438.1	99	SF174_1525
	ilvE	WP_035204145.1	100	SF174_1795
	coaX	WP_011244971.1	100	SF174_3555
	panB	WP_011246910.1	99.6	SF174_4003
	panC	WP_011246909.1	100	SF174_4004
	panD	WP_0112466908.1	100	SF174_4005
**B6**	*pdxR*	WP_035205374.1	100	SF174_0948
	pdxS	WP_011245312.1	100	SF174_0949
	pdxT	WP_011245313.1	100	SF174_0950
	pdxK	WP_035205840.1	100	SF174_2279
**K2**	*folC*	WP_035201802.1	100	SF174_0212
	folE	WP_011246726.1	100	SF174_1844
	phoA/B	WP_011248786.1	100	SF174_2259
	folA	WP_011247796.1	100	SF174_2916
	pabB	WP_051881120.1	100	SF174_3558
	pabA	WP_011244975.1	100	SF174_3559
	pabC	WP_035202184.1	100	SF174_3560
	folP	WP_035202185.1	100	SF174_3561
	folB	WP_035202207.1	99.2	SF174_3562
	folK	WP_011244979.1	100	SF174_3563
**Lipoic Acid**	*lipM*	WP_011247334.1	100	SF174_0347
	lipL	WP_035205862.1	100	SF174_2302
	lipL1	WP_011246457.1	100	SF174_2656
	lipA	WP_011247792.1	100	SF174_2912

Table S3. Complete and incomplete phage-elements found in SF106 and percentage of identity with proteins encoded by the reference strain *B. subtilis* 168.

**phage-like element**	**Product**	**Protein ID of *B. subtilis* 168**	**protein identity (%)**	**Target protein**
**complete**	ABC toxin/antitoxin/antitoxin system	NP_389166.1	100	SF106_1680
	antitoxin B	NP_389165.1	100	SF106_1681
	phage PBSX; N-acetylmuramoyl-L-alanine amidase	NP_389164.1	100	SF106_1682

phage PBSX; holin	NP_389163.1	100	SF106_1683
phage PBSX; putative enzyme	NP_389162.1	100	SF106_1684
xepA	AAA22642.1	100	SF106_1685
xkdX	CAA94045.1	100	SF106_1686
xkdW	CAA94044.1	100	SF106_1687
xkdV	CAA94043.1	100	SF106_1688
phage PBSX; conserved hypothetical protein	YP_003097716.1	98.9	SF106_1689
xkdU	CAA94042.1	99.5	SF106_1690
xkdT	CAA94041.1	100	SF106_1691
xkdS	CAA94040.1	100	SF106_1692
xkdR	CAA94039.1	100	SF106_1693
xkdQ	CAA94050.1	100	SF106_1694
xkdP	CAA94038.1	100	SF106_1695
xkdO	NP_389150.2	100	SF106_1696
xkdN	QJF44995.1	100	SF106_1697
xkdM	CAA94035.1	100	SF106_1698
xkdK	CAA94066.1	100	SF106_1699
xkdJ	CAA94065.1	100	SF106_1700
xkdI	CAA94064.1	100	SF106_1701
xkdH	CAA94063.1	100	SF106_1702
phage PBSX; conserved hypothetical protein	YP_003097713.1	100	SF106_1703
xkdG	CAA94062.1	99.7	SF106_1704
xkdF	CAA94061.1	98.9	SF106_1705
xkdE	CAA94060.1	99.8	SF106_1706
phage PBSX; prophage terminase (large subunit)	NP_389140.1	100	SF106_1707
phage PBSX; prophage terminase (small subunit)	NP_389139.1	100	SF106_1708
phage PBSX; putative RNA polymerase sigma factor-like	NP_389138.1	100	SF106_1709
xtrA	CAA94056.1	100	SF106_1710
xkdD	CAA94055.1	99.1	SF106_1711
phage PBSX; conserved hypothetical protein	YP_003097712.1	100	SF106_1712
xkdC	CAA94054.1	99.5	SF106_1713
xkdB	NP_389134.2	100	SF106_1714
phage PBSX; conserved hypothetical protein	YP_003097711.1	100	SF106_1715
xre	CAA84042.1	100	SF106_1716
phage PBSX; putative peptidase	NP_389132.3	99.5	SF106_1717
phage PBSX; manganese-containing peroxidase	NP_389131.2	100	SF106_1718
phage PBSX; gamma-polyglutamate hydrolase	NP_389130.1	100	SF106_1719
phage PBSX; conserved hypothetical protein	NP_389129.1	100	SF106_1720
phage PBSX; N-acetylmuramoyl-L-alanine amidase	NP_389128.1	99.7	SF106_1721
putative enzyme	NP_389127.2	100	SF106_1722
phosphatase RapA inhibitor PhrA	NP_389126.1	100	SF106_1723
Phage-like element PBSX protein XtrA	WP_003244900.1	38.5	SF106_0483
conserved phage protein of unknown function	NP_390500.1	100	SF106_0484
hypothetical protein; skin element	NP_390498.1	100	SF106_0485
hypothetical protein; skin element	NP_390498.1	100	SF106_0486
putative phage-related terminase small subunit; skin element	NP_390497.1	100	SF106_0487
Phage terminase large subunit	AIY95529.1	74.5	SF106_0488
putative phage capsid protein; skin element	NP_390495.1	63.9	SF106_0489
putative phage head morphogenesis protein; skin element	NP_390494.1	100	SF106_0490
conserved phage protein of unknown function	NP_390493.1	100	SF106_0491
Putative phage serine protease XkdF	CAA94061.1	62	SF106_0492
putative phage capsid protein	NP_390491.2	80.4	SF106_0493
hypothetical protein; skin element	NP_390490.1	99	SF106_0494
conserved phage protein of unknown function	NP_390489.2	50.9	SF106_0495

incomplete	conserved phage protein of unknown function	NP_390488.1	54.3	SF106_0496
	putative phage tail component	NP_390487.1	51	SF106_0497
	conserved phage protein of unknown function	NP_390486.2	52.1	SF106_0498
	conserved phage protein of unknown function	YP_003097763.1	54.7	SF106_0499
	putative phage tail sheath protein	NP_390485.2	100	SF106_0500
	putative tail tube protein	NP_390483.2	95.2	SF106_0501
	toxic peptide of toxin-antitoxin system; skin element	NP_390482.1	100	SF106_0502
	phage portal protein	P45930.2	85.9	SF106_0503
	putative tape measure protein; skin element	NP_390480.2	100	SF106_0504
	putative phage murein-binding protein; skin element	NP_390479.1	83	SF106_0505
	conserved phage protein of unknown function	NP_390478.1	100	SF106_0506
	conserved phage protein of unknown function	NP_390477.1	100	SF106_0507
	conserved phage protein of unknown function	NP_390476.1	85.8	SF106_0508
	putative phage baseplate assembly protein	NP_390475.1	88.8	SF106_0509
	putative phage tail baseplate protein	NP_390474.2	86.9	SF106_0510
	conserved phage protein of unknown function	NP_390473.1	76.7	SF106_0511
	phage PBSX; conserved hypothetical protein	NP_389158.1	79.5	SF106_0512
	conserved phage protein of unknown function	NP_389159.1	66.3	SF106_0513
	conserved phage protein of unknown function	NP_390470.1	68.5	SF106_0514
	putative phage-related lytic exoenzyme	NP_390469.1	56	SF106_0515
	putative holin; skin element	NP_390468.1	100	SF106_0516
	N-acetylmuramoyl-L-alanine amidase; skin element	NP_390467.1	64.7	SF106_0517
	hypothetical protein; skin element	NP_390466.1	100	SF106_0518

Table S4. Complete and incomplete phage-elements found in SF174 and percentage of identity with proteins encoded by the reference strain *A. clausii* ENTPro.

phage-like element	Product	Protein ID of *A. clausii* ENTPro	protein identity (%)	Target protein
complete	terminase small subunit	WP_035201295.1	100	SF174_4112
	PBSX family phage terminase large subunit	WP_035201291.1	100	SF174_4113
	phage portal protein	WP_035201276.1	100	SF174_4114
	phage head morphogenesis protein	WP_035201273.1	100	SF174_4115
	hypothetical protein	WP_035201270.1	100	SF174_4116
	DUF4355 domain-containing protein	WP_051881081.1	100	SF174_4117
	hypothetical protein	WP_035201266.1	100	SF174_4118
	major capsid protein	WP_035201263.1	100	SF174_4119
	hypothetical protein	WP_156323132.1	100	SF174_4120
	phage head-tail connector protein	WP_035201260.1	100	SF174_4121
	hypothetical protein	WP_035201257.1	100	SF174_4122
	HK97 gp10 family phage protein	WP_094978930.1	100	SF174_4123
	DUF3168 domain-containing protein	WP_035201251.1	100	SF174_4124
	phage major tail protein, TP901-1 family	WP_051881080.1	100	SF174_4125
	hypothetical protein	WP_051881079.1	100	SF174_4126
	hypothetical protein	WP_051881078.1	98.7	ND
	hypothetical protein	WP_151210677.1	99.9	SF174_4127
	phage tail family protein	WP_035201248.1	100	SF174_4128
	phage tail protein	WP_035201246.1	100	SF174_4129
incomplete	helix-turn-helix transcriptional regulator	WP_142300536.1	100	ND
	hypothetical protein DB29_04200	ALA55028	97.7	ND
	helix-turn-helix domain-containing protein	WP_035201332.1	100	SF174_0758
	hypothetical protein	WP_035201329.1	100	SF174_0759
	hypothetical protein	WP_156323130.1	100	SF174_0760

hypothetical protein	WP_035201326.1	100	SF174_0761
hypothetical protein	WP_051881085.1	100	SF174_0762
hypothetical protein	WP_169024188.1	100	SF174_0763
hypothetical protein	WP_051881084.1	100	SF174_0764
DUF6011 domain-containing protein	WP_035201322.1	98.6	SF174_0766
hypothetical protein	WP_035201320.1	100	SF174_0767
single-stranded DNA-binding protein	WP_035201318.1	100	SF174_0768
hypothetical protein	WP_035201316.1	100	SF174_0770
Holliday junction resolvase RecU	WP_035201314.1	100	SF174_0771
DNA adenine methylase	WP_035201312.1	100	SF174_0772
BRO family protein	WP_051881083.1	100	SF174_0773
XtrA/YqaO family protein	WP_035201311.1	100	SF174_0774
hypothetical protein DB29_04216	ALA55044.1	97.7	ND
sigma-70 family RNA polymerase sigma factor	WP_035201304.1	100	SF174_0777

ND= Not Determined

Table S5. Genes associated to antibiotic resistance present in the SF106 or SF174 genome.

Antibiotic	Query	Description	locus
**Streptomycin**	SF106_0428	aminoglycoside 6-adenylyltransferase	core
**Chloramphenicol**	SF174_0915	Small Multidrug Resistance protein	core
	SF174_1140	Arabinose efflux permease	core
	SF174_2789	Methylates position 8 of adenine 2503 in 23S rRNA.	accessory(12/14)
	SF174_4186	effector of chloramphenicol resistance in bacteria	accessory (9/14)
**Erythromycin**	SF174_1556	Erythromycin esterase	core
	SF174_2668	Erythromycin esterase	accessory(12/14)
**Clindamycin**	SF174_1582	Ribosomal RNA adenine dimethylases	core
**Kanamicin**	SF174_3794	Protein of unknown function (DUF1679)	core
**Streptomycin**	SF174_1377	Streptomycin adenylyltransferase	core
	SF174_1427	Streptomycin adenylyltransferase	core
	SF174_1758	Mediates bacterial resistance to the antibiotics streptomycin	accessory (9/14)
**Tetraciclin**	SF174_0916	Trenscriptional regulator	core
	SF174_1786	Trenscriptional regulator	core
	SF174_3373	Elongation factor G, domain IV	core
**Vancomycin**	SF174_1636	VanW like protein	core

Figure S1: Organization of the gene cluster involved in the production of vitamins in SF106 (left) and SF174 (right).

Figure S2: Organization of the antimicrobial peptides biosynthetic gene cluster in SF106 (**A, B** and **C**) and SF174 (**D**).
