# Supplementary figures and images for: Comparative Genomics and Physiological Characterization of Two Aerobic Spore Formers Isolated from Human Ileal Samples
Source: Int J Mol Sci. 2022 Nov 29;23(23):14946. doi: 10.3390/ijms232314946 (PMC9739757; doi:10.3390/ijms232314946)

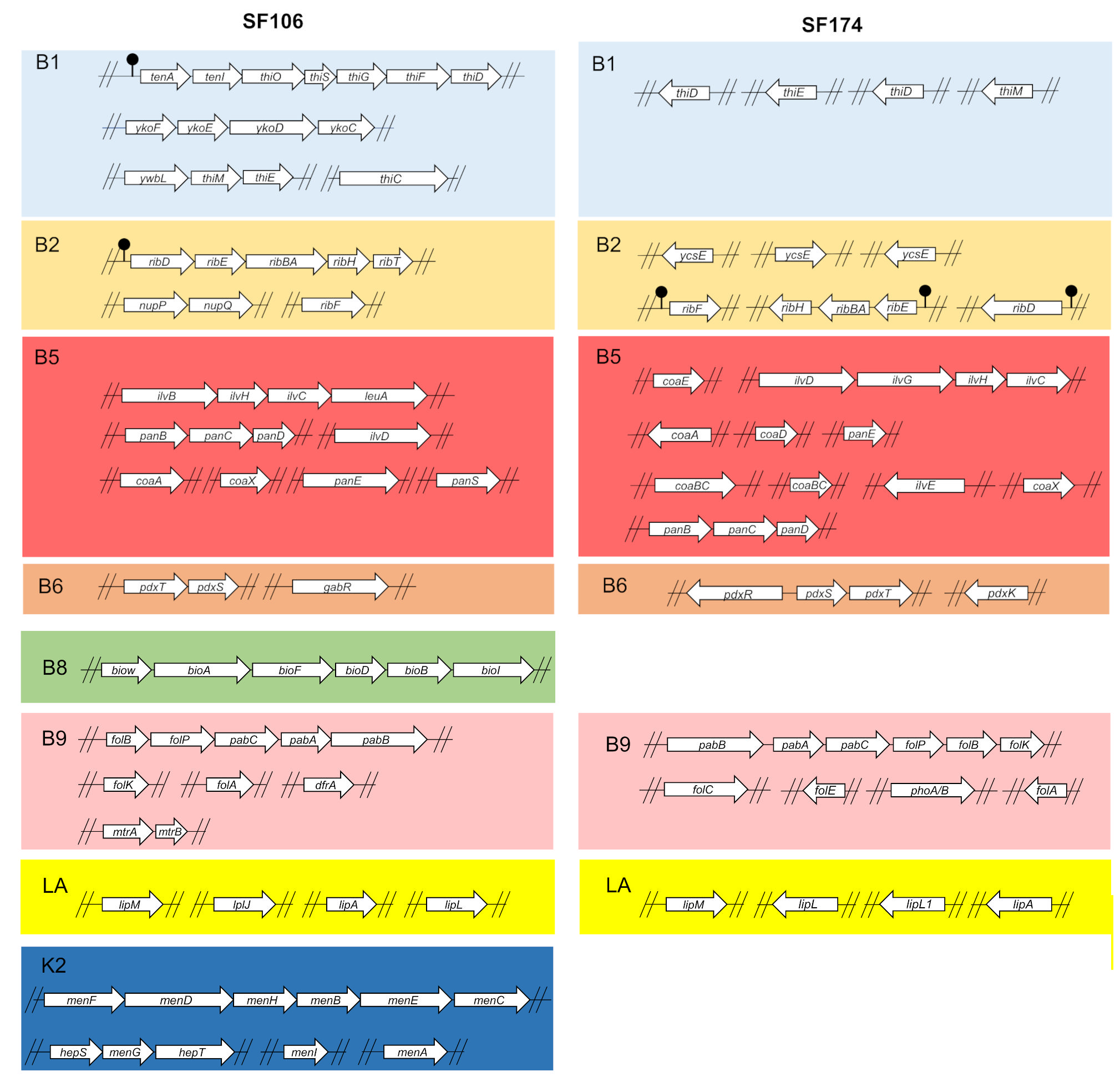

Supplement: Supplementary file 1 [file ijms-23-14946-s001.zip › figures s1.tif]

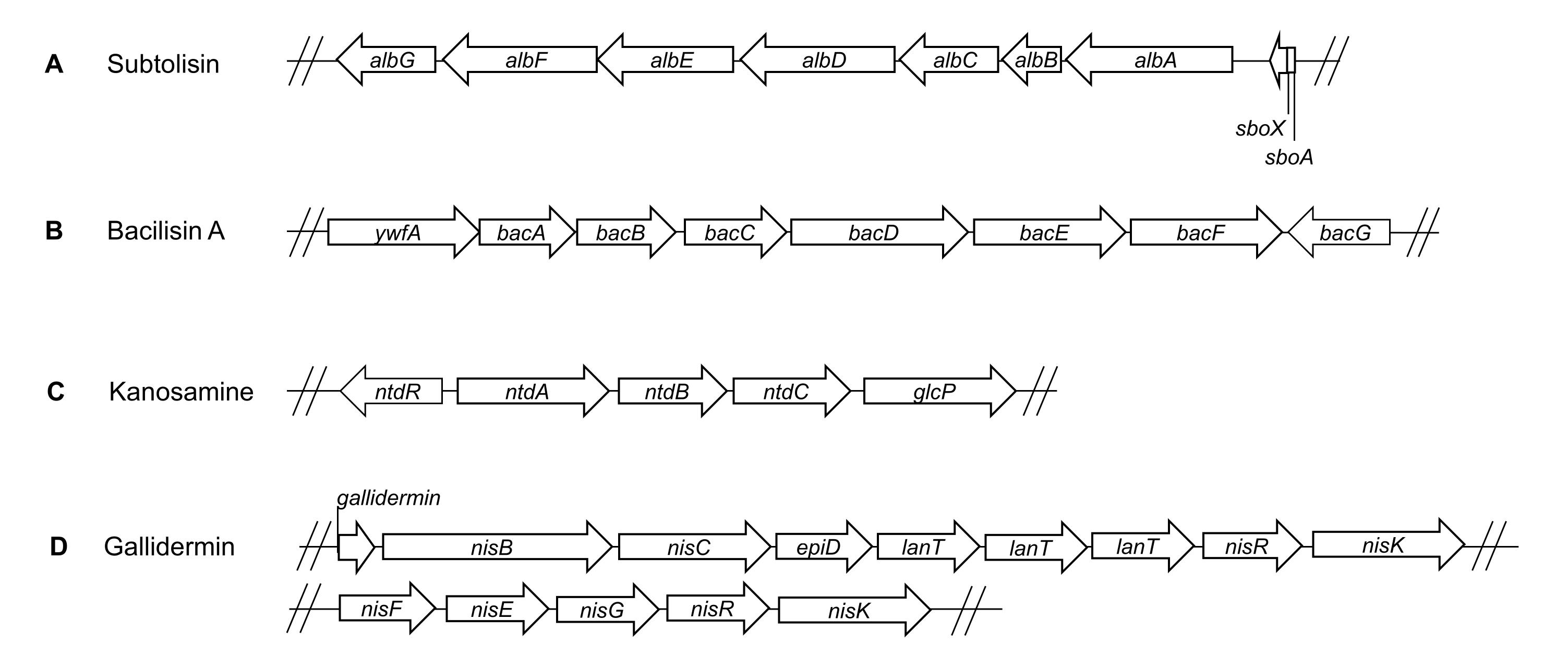

Supplement: Supplementary file 1 [file ijms-23-14946-s001.zip › figures s2.tif]
